# Uncertainty about flying conspecifics causes territorial contests of the Old World swallowtail, Papilio machaon

**DOI:** 10.1186/s12983-019-0324-y

**Published:** 2019-06-24

**Authors:** Tsuyoshi Takeuchi, Shinji Yabuta, Hiroyuki Takasaki

**Affiliations:** 1 Entomological Laboratory, Graduate School of Life and Environmental Sciences, Osaka Prefecture University, 1-1 Gakuencho, Nakaku, Sakai, Osaka, 5998531 Japan; 2 Department of Animal Sciences, Teikyo University of Science & Technology, 2525 Yatsusawa, Uenohara, Yamanashi, 4090193 Japan; 3 Department of Zoology, Faculty of Science, Okayama University of Science, Ridai-cho, Okayama, Okayama 7000005 Japan

**Keywords:** Butterfly, Courtship, Mating system, Sexual recognition, Sexual selection, Territory, War of attrition

## Abstract

**Background:**

Male-male aerial contests of territorial butterflies are difficult to explain by major contest models based on game theory because of butterflies’ apparent inability to inflict substantial costs on their opponent. As an alternative, the “erroneous courtship hypothesis” was presented. This hypothesis is based on the assumption that territorial butterflies cannot discriminate the sex of flying conspecifics. The hypothesis regards the aerial contest of male butterflies as an inevitable same-sex entanglement in the butterflies’ behavioral sequence. To test the robustness of the hypothesis, we investigated the sex recognition abilities of the Old World swallowtail, *Papilio machaon*.

**Results:**

We presented four types of flapping butterfly specimens (fresh male and female, chemicals-removed male and female) to territorial males. The males touched fresh female specimens and showed typical courtship flight. For the other types of specimens, they rarely showed courtship flight although they approached or touched them. In addition, territorial males reacted longer to fresh males than to fresh females.

**Conclusions:**

The results indicated that although territorial males recognize flying females as sexual partners by sensing their semiochemicals, they cannot identify flying conspecific males, and continue to gather information on them. *P. machaon* is one of the species whose behavior is most incompatible with the erroneous courtship hypothesis, as its males perform a typical courtship flight to flying females, suggesting the ability of sexing flying conspecifics. Nevertheless, the erroneous courtship hypothesis was not disproved by our results.

**Electronic supplementary material:**

The online version of this article (10.1186/s12983-019-0324-y) contains supplementary material, which is available to authorized users.

## Background

Male-male contests over mating opportunities are ubiquitous in the animal kingdom [1]. Physical attack sometimes occurs, attended by the risk of serious injury or death. Contest models based on game theory predict that the outcome of such violent contests is usually settled by asymmetry in resource holding potential (RHP; sensu [2]). In theoretical terms, individuals with higher RHP can inflict greater costs on their opponent and minimize their own cost accrual. RHP is usually correlated with morphological structures, e.g., body size or weaponry [3]. However, not all animals show such morphological adaptations. Butterflies are typical examples. They have neither weapons nor organs useful for injuring their opponents (e.g., teeth, nails or horns). Nevertheless, males of various butterfly species occupy a mating territory, located at hilltops, forest edges, or sunspots in forests. They compete over the territory through aerial interactions [4]. Typically, a territorial male rushes toward an intruding male. Then the two males fly around each other until one of them retreats. During such aerial interactions, no apparent attack is made. Therefore, it is difficult to estimate the costs imposed on the contestants. Consequently, butterfly contests have often been interpreted using war of attrition models [4]. In these models, contestants perform displays that impose costs (e.g., energy) not on their opponent but on themselves. One of the contestants retreats when the accumulated cost reaches its threshold [5, 6].

Although war of attrition models are well known to behavioral ecologists, the existence of wars of attrition in the real world is open to doubt. In a general model of prolonged display in animals, Payne [7] pointed out the following two features. (1) One particular requirement in wars of attrition is the need for matching the intensity of the two contestants’ displays. (2) When such matching is not enforced, one consequence is inviting cheats who delay (or stop) their own display in order to gain an energetic advantage in any subsequent fight. In a review of past research on territorial butterflies, Takeuchi et al. [8] found no report of such enforcement, e.g., attacks on non-displaying contestants by their opponent. There is also no evidence of female preference for males that perform aerial contests. When two individuals compete over a mating territory under this condition, both contestants should stay in the territory without displaying to wait for the opponent to leave. Even if the opponent will not leave, there is no way for a male to force the opponent to leave because he cannot impose costs on the opponent. At present, the framework of wars of attrition cannot explain why territorial butterflies should perform aerial contests. In addition, further difficulties arise owing to the limitations of butterflies’ cognitive abilities. To date, there is no evidence that territorial butterflies recognize the sex of their opponent during their aerial contests [8]. This is problematic, as major contest models based on game theory (hereafter, major contest models) assume that contestants can distinguish rivals from others such as potential partners [5–7, 9] (but see Yabuta [10]).

As an alternative to major contest models, including wars of attrition, Takeuchi et al. [8] presented another framework to understand butterfly contests, i.e. “the erroneous courtship hypothesis”. This hypothesis assumes that males cannot discriminate the sex of flying conspecifics, and have some uncertainty about the species of flying objects. Territorial interactions occur as two males chase each other, expecting their opponent to be a receptive female. At last, one of them gives up chasing the opponent. Then this male flees from the opponent, which is advantageous because it may be a natural enemy. The erroneous courtship hypothesis follows the principle of parsimony: entities must not be multiplied beyond necessity. In the present case, the ability to perform sexual (and species) identification must not be assumed unless some facts remain inexplicable without assumption of this ability. In contrast to the major contest models, the present hypothesis can explain why territorial butterflies perform aerial displays. Note that the erroneous courtship hypothesis considers aerial interactions of territorial butterflies to function as contests in that the owner of an indivisible resource is determined through the behavior. However, it does not regard the aerial interactions as agonistic displays. Since the erroneous courtship hypothesis does not assume their ability to discriminate the sex (and species) of flying conspecifics, it is based on simpler cognitive assumptions than major contest models [8, 11]. If facts remain inexplicable without the assumption that territorial males know that their opponent is a conspecific male (a potential sexual rival) during their aerial interactions, the erroneous courtship hypothesis does not hold true. This hypothesis is thus falsifiable.

**Fig. 1:**
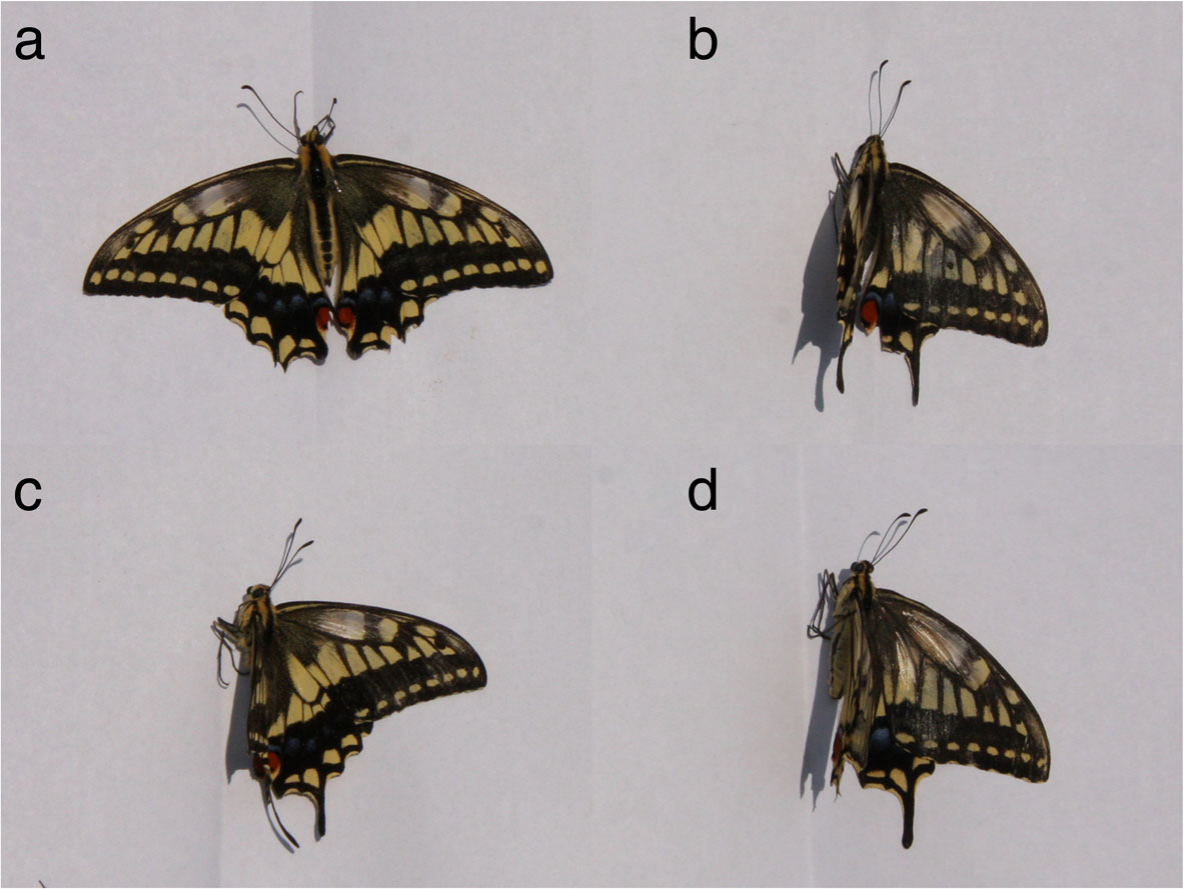
The four types of butterfly specimens used in the present study. **a** fresh male; **b** fresh female; **c** treated male; **d** treated female

The Old World swallowtail, *Papilio machaon* (Lepidoptera: Papilionidae), has a territorial mating system [12, 13]. Its males perform a typical courtship flight to flying females before copulation, and during a male-male aerial interaction they sometimes grab their opponent with their legs [13]. Although *P. machaon*’s apparent sexual dimorphism is weak (Fig. 1), the typical courtship flight and male-male interactions of this species suggest that its males possess the ability of sexing flying conspecifics. Therefore, this species is an ideal experimental candidate for disproving the erroneous courtship hypothesis. If its males can identify flying conspecific males as males (potential sexual rivals), the hypothesis must be discarded, like a null hypothesis in statistical tests. If, however, the males cannot do such sexing, the hypothesis fulfills the principle of parsimony.

It is relatively easy to demonstrate that males recognize something as a female because one can use a sequence of mating behavior as evidence. If one demonstrates that males recognize something as a male, the typical evidence is an attack or threat to the thing when it approaches his mating chances (e.g., [14]). Although it is not known that male butterflies exhibit such behavior (this is why the erroneous courtship hypothesis works better than major contest models for butterflies), *P. machaon* is one of the species with the highest probability of exhibiting such behavior. This study attempted to reject the key assumption of the erroneous courtship hypothesis that territorial males cannot identify the flying conspecific males as being males. We prepared two types of specimens of both sexes, fresh ones and treated (chemical-removed) ones (Fig. 1). These specimens were flapped using a motor to imitate flying butterflies, and were presented to territorial males of *P. machaon* to compare male responses to each model. If territorial males attack or threaten the male specimen preferentially, the erroneous courtship hypothesis should be discarded, and major contest models should be applied.

### Definitions and terminology

We define the recognition of the same sex (male) as recognition of a sexual rival, and the recognition of the opposite sex (female) as recognition of a sexual partner. For clear discussion, distinction of butterflies’ ‘recognition’ from our human observers’ recognition is indispensable. Therefore, when we refer to the butterflies’ ‘recognition’, we use single quotation marks. For example, the results showed that the male butterfly recognized the female lure as a ‘female’ because he attempted to copulate with it.

## Results

**Fig. 2:**
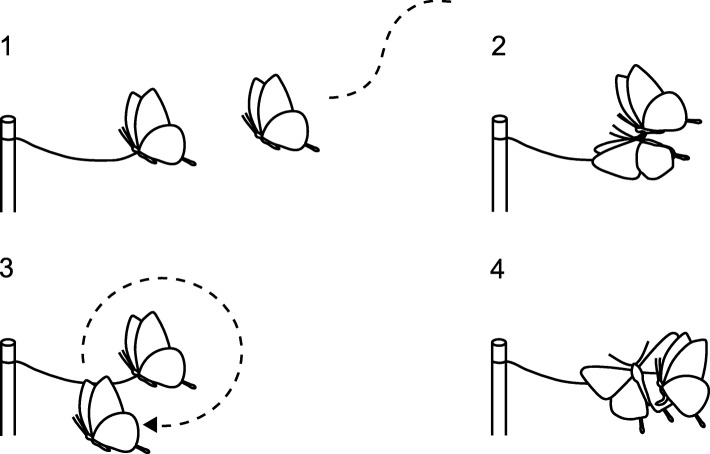
Male response phases. 1. approach; 2. touch; 3. courtship flight; 4. copulation attempt. Males that exhibited behaviors of later phases also exhibited those of earlier phases (but the reverse was not always true)

Responses of territorial males to flapping specimens were scored as four sequential phases (Fig. 2): (1) approach: change flight direction to the specimen; (2) touch: touch the wings of the specimen with their legs; (3) courtship flight: perform the specific flight (described in Study species and sites) to the specimen; (4) copulation attempt: bend its abdomen to that of the specimen. The behavioral repertoires performed toward male specimens were a part of those performed toward female specimens. We regarded their courtship phases to progress in this order because the territorial males that exhibited behaviors of later phases also exhibited those of earlier phases (but the reverse was not always true).

Territorial males approached all types of flapping specimens. When the specimen was treated (chemicals-removed), some males flew away, and the others touched the wings of the specimen (often at the base of the forewing). When the specimen was a fresh male, most territorial males continued to touch its wings. Two territorial males reacted to a fresh male for more than 10 min. When the specimen was a fresh female, they touched its wings and showed courtship flight. After they reacted to the flapping specimens, they resumed normal territorial behavior. Videos of male responses to the fresh specimens are included in Additional file**s** 1 and 2.

**Fig. 3:**
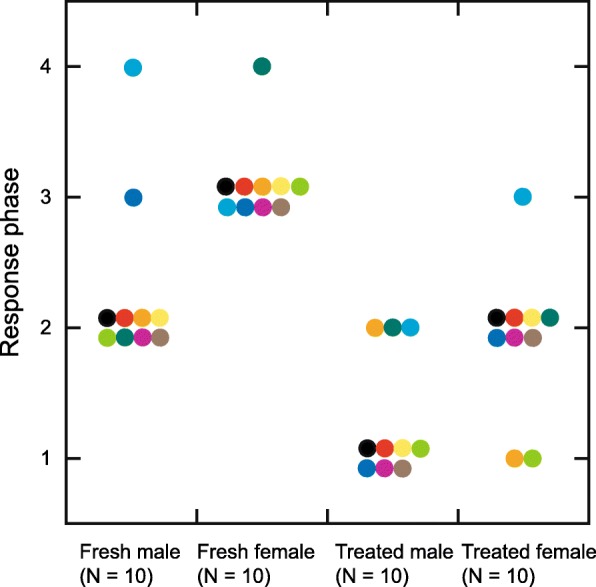
Male responses to each specimen. Different colors indicate different individuals

The mixed effect model analyzing the maximum response phases showed significant effects (Table 1). Territorial males showed more advanced response phases to the females and fresh specimens than to the males and treated specimens (Fig. 3; Additional file 3).

**Table 1:** The coefficients of the ordered logistic regression with random effects

	Estimate	Std. Error	z value	Pr (>|z|)
Sex	−3.2	1.3	−2.5	0.0012
State	6.2	1.9	3.3	0.0011
Sex*state	−0.83	1.7	−0.48	0.63

**Fig. 4:**
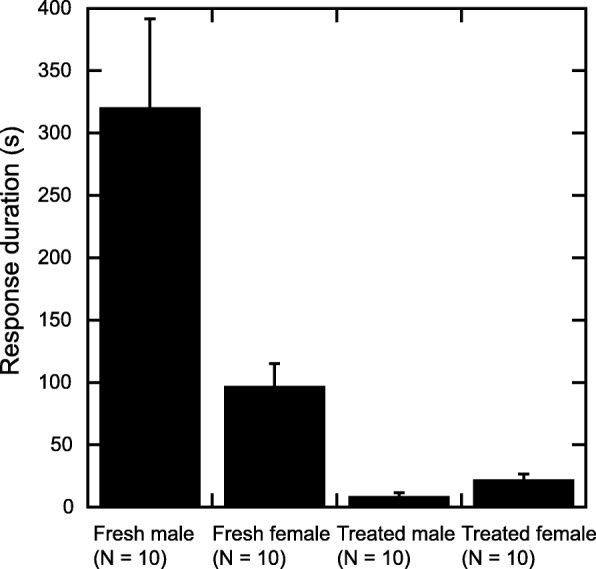
Duration of male response to each specimen. Mean and s.e. of original data are shown

The mixed effect model analyzing response duration also showed significant effects (Table 2). Territorial males reacted longer to fresh specimens than to treated specimens (Fig. 4; Additional file 3). Interestingly, they reacted longer to treated females than to treated males, whereas they reacted longer to fresh males than to fresh females (Fig. 4).

**Table 2:** The coefficients of the mixed effect Cox model

	coef	Exp (coef)	Se (coef)	z	P
Sex	1.1	3.0	0.52	2.1	0.034
State	−2.5	0.083	0.72	−3.5	0.00049
Sex*state	−2.8	0.060	0.80	−3.5	0.00041

## Discussion

Territorial males showed various response phases to the flapping specimens (Fig. 3). At first, they approached the specimen. They exhibited more advanced phases toward fresh specimens than treated specimens. When the specimen was a fresh male, they continued to touch its wings. When the specimen was a fresh female, they touched its wings and showed courtship flight. We found no specific behavior toward male specimens. Here we recall the principle of parsimony: the ability of sexual identification should not be assumed unless facts remain inexplicable without assuming the ability. The simplest interpretation of the results is as follows. At first, males visually find flying conspecifics, and then they touch their wings to detect semiochemicals. If the specimen is a fresh female, they recognize it as a ‘female’ and start courtship flight. If the specimen is a fresh male, they cannot ascertain whether it is a ‘female’ and continue to stay in this stage. If the specimen is treated (chemicals-removed), males easily judge that it is not a ‘female’. Although the original erroneous courtship hypothesis assumed that male butterflies cannot sex flying conspecifics [8], males of *P. machaon* identify flying conspecific females. However, it is difficult for them to judge what a flapping fresh male is. Hence, they continue to investigate what it is. Thus, the erroneous courtship hypothesis was not disproved, because the hypothesis is applicable to male-male contests [8]. Note that we do not rule out the possibility that butterflies can recognize conspecific males as a ‘male’ during their aerial interactions and perform wars of attrition or other types of contest behavior [7, 9]. Based on the principle of parsimony, however, we state that the assumption of this ability and interpretations based on it are unfounded and unnecessary at present.

In addition, our results indicated that males identify ‘females’ by semiochemicals, although it is reported that there is no female-specific volatile chemical compound of *P. machaon* [15]. Interestingly, although territorial males showed more advanced courtship phases toward fresh females than toward fresh males, they reacted longer to fresh males than to fresh females (Fig. 4). The length of responses may not be an indicator of preference, but rather an indicator of a delay in decision making.

We should state the limitations of our experiments. The flapping specimens did not react to territorial males, whereas real butterflies do. In addition, the chemical-removed specimens might differ from fresh specimens not only in the amount of semiochemicals but also in other factors such as loss of scales. These differences might hinder territorial males from identifying a ‘conspecific male’. However, no research has demonstrated that sexual recognition of male butterflies is based on such subtle differences.

Male butterflies generally respond to a conspecific by visual cues at first, and then at close contact, they use chemical information to confirm whether it is a ‘female’ [16–20]. Note that even if males are more attracted to females than to males, this does not necessarily mean that males can identify the males’ sex. Kato and Yoshioka [18] reported an interesting example in this regard. Namely, although almost all males of the blue triangle, *Graphium sarpedon*, tried to copulate with fresh female specimens, more than half of them tried to copulate with fresh male specimens too.

In male-male interactions of *P. machaon*, the two males often ascended with spiral flight, and touched or grabbed the opponent with their legs [13]. It was also reported that males of *P. indra* and *P. zelicaon* compete by locking legs and beating their wings [21, 22]. These two species are closely related to *P. machaon* [23]. Our findings in the present experiments strongly suggest that the male-male locking in their contest is aimed at obtaining semiochemicals to judge whether their opponent is a ‘female’. In contrast to the behavior observed in our experiments, wild males lock the opponent’s legs in their interactions. This is because in natural male-male interactions, both males are likely to attempt to touch the opponent’s wings, whereas in our experiments the flapping specimen did not react to the territorial male. Also in a related species, *P. xuthus*, males attracted to a female touch her wings with their legs [16].

## Conclusions

Butterflies do not have functional weapons. This fact seems incompatible with the possibility that they have contest behavior. Nevertheless, territorial butterfly males determine the owner of a mating territory through aerial interactions [4]. The present study supports the framework that aerial contests of the butterflies occur as they do not know what their opponent is. In other words, uncertainty about their flying opponent enables their territorial mating system to function. Although aerial interactions of male butterflies are unlikely be adaptive behavior, the owner of a mating station is determined through the interaction. What kind of individuals become an owner? The erroneous courtship hypothesis assumes that territorial butterflies do not precisely know the species of their opponent [8]. Therefore, their opponent may be a natural enemy. Under this condition, more fearless individuals may chase the opponent longer and become an owner. The evolutionary sequence of their aerial interactions should be investigated to look for insight into this possibility.

Butterflies are not the only taxon that performs aerial contests. It is well known that territorial odonata perform aerial contests [24]. The sexual discriminative abilities of odonate males vary among species [8]. Ishizawa and Arai [25] reported that the territorial dragonfly *Anotogaster sieboldii* responded to both fluttering males and females, or even to rotation of a mini desk fan. They suggested that the observed aerial interactions are behavior to ascertain the opponent’s sex. The applicability of the erroneous courtship hypothesis to flying insects other than butterflies is still an open question.

## Methods

### Study species and sites

*Papilio machaon* is the type species of the genus *Papilio*, and is widely found in the Palearctic region and North America [26]. Its large size and widespread distribution make it an ideal butterfly species for scientific research. In Japan, it (subsp. *P. m. hippocrates*) is common with no call for any law for its protection. Adults appear from spring to autumn in Japan. Takeuchi [13] reported the mating behavior of *P. machaon*. In daytime, males gather on hilltops, where they hold a mating territory. When another male flies into the territory, the territorial male rushes to him. Then, the two males perform aerial contests. When a female flies into the territory, the territorial male chases her to catch up. He often courts the flying female by following her from behind. Then he flies just beneath her before flying up in front of her, and drops back behind her to loop his flight routine again. During this process, the male sometimes contacts her with his legs. If the female alights nearby, the male also alights by her and they copulate.

Larvae of the butterfly were collected on the campus of Osaka Prefecture University, Sakai, Osaka Pref., Japan, in May 2017. We gave them leaves of Japanese parsley, *Oenanthe javanica*, at room temperature. When adults emerged, each was stored in an entomological envelope at 12 °C until field experiments. They were fed 5% sugar solution every other day. These butterflies were used to make flapping specimens.

Field experiments were performed at Mt. Ohyasan (754 m a.s.l.), Inagawa Town, Hyogo Pref., Japan, between June 22 and August 4, 2017. The hilltop was grassy, and territorial males of *P. machaon* perched on the ground.

### Experimental procedures

We imitated flying butterflies using motor-driven specimens as described (and illustrated) by Takasaki et al. [27]. The device resembles that used by Stoehr et al. [28] for the cabbage white, *Pieris rapae*. In our device, a butterfly specimen was mounted on a piece of thin piano wire driven by a DC motor. The body of the specimen was fixed at one end of the piano wire (ca. 25 cm long). The other end of the wire was connected to a disc through a small loose hole at a distance of ca. 1 cm from the center of the disc. The disc was mounted at its center on the shaft of the motor. As the motor runs, the rotation is transmitted to the wire through the joint via a kind of crank mechanism. The rotation of the motor and the resilience of the wire drive the specimen up-and-down. The up-and-down motion makes the specimen flap its wings, in particular when using fresh specimens. The flapping speed is regulated by changing the voltage supplied to the motor.

As *P. machaon* flaps at ca. 13 Hz (TT, unpubl. data), our motor’s rotation cycle (ca. 15~20 Hz) was slightly faster than the real butterfly’s flapping cycle. This was an unavoidable discrepancy, because when the motor was rotated at ca. 13 Hz, it often stopped when a stimulated male touched and landed on the specimen. We used four types of butterfly specimens (Fig. 1): (a) fresh male; a specimen killed within 3 h before the experiment; (b) fresh female; prepared similarly; (c) treated male; a specimen soaked in chloroform for 24 h to remove chemicals, and then rinsed with fresh chloroform for 24 h; (d) treated female; prepared similarly.

Ten wild territorial males of *P. machaon* were used for our experiments. We presented the four types of flapping butterfly specimens, one at a time, to each territorial male, and video-recorded (Everio GZ-E765, JVCKENWOOD) the reactions of these males. We stopped recording when the male flew away from the specimen or reacted to the specimen for longer than 10 min. The order of presenting the four types of specimens was determined randomly, and changed for each territorial male. Males were considered “territorial” when they chased an intruding object, and returned to the hilltop. When more than one male was present around the hilltop, we retained one but captured and removed the others from the immediate vicinities before the experiment. Otherwise they often came together to the flapping specimen, and made it unclear whether the focal male reacted to the specimen or to the other males. Territorial males used for our experiments were individually marked with water-insoluble ink to make it possible to obtain independent data.

### Data analyses

Two mixed effect models were constructed to analyze the male responses. The maximum response phase was analyzed using an ordered logistic regression with random effects. The sex and state (fresh or treated) of the specimen and their interactions were included as independent variables. Each individual was included as a random factor. For this analysis, we used the clmm function in the package ordinal [29] for R 3.4.1 [30]. Response duration was analyzed using a mixed effect Cox model. The sex and state (fresh or treated) of the specimen and their interactions were included as independent variables. Each individual was included as a random factor. Responses lasting longer than 10 min were treated as truncated data. For this analysis, we used the coxme function in the package coxme [31] for R 3.4.1 [30].

## Additional files

Additional file 1: A response of a territorial male to a fresh male (phase 2). (MP4 6682 kb)

Additional file 2: A response of a territorial male to a fresh female (phase 3). (MP4 6612 kb)

Additional file 3: Data of response phase **/** duration (s) to each specimen. (XLSX 10 kb)

## Data Availability

Data of response phase and duration are presented in Additional file 3.
